# Salvage surgery for advanced non‐small cell lung cancer after targeted therapy: A case series

**DOI:** 10.1111/1759-7714.13366

**Published:** 2020-02-28

**Authors:** Weian Song, Shouyin Di, Junqiang Liu, Boshi Fan, Jiahua Zhao, Shaohua Zhou, Siyu Chen, Hai Dong, Caiying Yue, Taiqian Gong

**Affiliations:** ^1^ Department of Thoracic Surgery The Sixth Medical Center of Chinese PLA General Hospital Beijing China

**Keywords:** Non‐small cell lung cancer, salvage surgery, survival, targeted therapy

## Abstract

**Background:**

Tumor recurrence or residual tumor after targeted therapy is common in patients with advanced non‐small cell lung cancer (NSCLC). There is a lack of high‐level evidence on which type of treatment should be employed for these patients and the role of salvage surgery has not been well reported in the literature.

**Methods:**

A retrospective analysis of patients who underwent salvage surgery in our center between January 2016 and June 2019 for advanced NSCLC after targeted therapy was performed.

**Results:**

A total number of nine patients were identified, including five males and four females, with a median age of 56 years (range, 40–65 years), all diagnosed with lung adenocarcinoma stage IIIa–IVb. All patients had received targeted therapy according to individual positive mutation of driver gene(s). Salvage surgery was performed for tumor recurrence or residual tumor after a duration of 2–46 months of targeted therapy. A negative surgical margin was achieved in all cases. Postoperative complication rate was 11.1% (1/9). All patients were alive at the time of this analysis and two patients had disease progression. After a median follow‐up of 17 months (range: 5–44 months), the median event‐free survival and postoperative survival was 14 months (range: 2–44 months) and 17 months (range: 5–44 months) respectively.

**Conclusions:**

Salvage surgery may be a feasible and promising therapeutic option for tumor recurrence or residual tumor in advanced NSCLC in selective patients after targeted therapy.

**Key points:**

- Salvage surgery is feasible in selected patients with advanced NSCLC and provides promising survival outcomes after targeted therapy failure.
- Salvage surgery provides precise molecular and pathological information which is most important for subsequent therapy.

## Introduction

Lung cancer is the most common malignancy and is the first killer of cancer‐related death worldwide.^1^ Nearly 85% of lung cancers are non‐small cell lung cancer (NSCLC). To date, surgery is still the cornerstone of NSCLC treatment strategy since it provides the opportunity of cure. Unfortunately, 70%–85% of NSCLC patients are at an advanced stage at the time of diagnosis and thus lose the opportunity of radical surgery.^2^ For patients with advanced NSCLC, platinum‐based chemotherapy, with or without local radiotherapy, is usually administered as first‐line treatment, although effectiveness is far from satisfactory. Five‐year survival rates of patients with stage III or IV diseases treated by chemotherapy and radiotherapy are only 30% and 5%, respectively.^3^

In the previous two decades, targeted therapy which employs a new class of drugs that specifically target certain molecular pathways has developed remarkably and dramatically changed the treatment strategy of lung cancer.^4^ Many patients with advanced lung cancer have benefited from targeted therapy with longer survival, fewer adverse effects and a better quality of life, compared with conventional chemotherapy and radiotherapy.^5^, ^6^ With the developments in molecular biology, targeted drugs are being constantly improved and updated to provide better therapeutic effect for lung cancer.^7^ However, tumor recurrence or residual tumor during targeted therapy has been commonly reported^6^, ^8^ and there is a lack of high‐level evidence on which type of treatment should be employed for these patients.^9^, ^10^ Conventionally, alternative medical treatment or radiotherapy may be administrated as second‐ or third‐line therapy, although the effect is usually unpredictable and poor.^11^, ^12^, ^13^ On the other hand, there are some patients having had their NSCLC converted from an advanced to resectable stage as a result of targeted therapy, that salvage surgery may be another alternative option for tumor recurrence or residual tumor after targeted therapy.^14^

Salvage surgery already plays a role in the treatment of esophageal cancer, colorectal cancer and other tumors.^15^, ^16^, ^17^ In recent years, salvage surgery has also been introduced into the treatment strategy of advanced lung cancer after chemotherapy/radiotherapy failure.^18^, ^19^, ^20^, ^21^, ^22^ In a study conducted by Hidetaka *et al*. a complete pathological response was found in nearly half the cases who received salvage surgery after chemotherapy/radiotherapy.^23^ However, the role of salvage surgery in patients with advanced lung cancer after targeted therapy has still not been well reported in the literature.^24^, ^25^

Here, we present our single‐center study and analyze the feasibility and efficiency of salvage surgery for advanced NSCLC after targeted therapy.

## Methods

### Study design and patients

All consecutive NSCLC patients receiving surgery after targeted therapy in the sixth medical center of the Chinese PLA General Hospital between January 2016 and June 2019 were collected. Patients were eligible for inclusion if they met the following criteria: (i) Pathologically identified NSCLC in clinical TNM stage of IIIa–IVb at the time of diagnosis, according to the eighth edition of the TNM staging system^26^; (ii) targeted therapy had been administered according to the positive mutation of corresponding driver gene(s), and (iii) comprehensive preoperative reassessment had been performed. Patients undergoing surgical biopsy for diagnostic purposes were excluded.

Demographic and clinical data including gender, age, clinical stage, histology, driver‐gene status, targeted therapy agents, duration of targeted therapy before surgery, surgical procedure, operation time, intraoperative blood loss, postoperative complications, length of hospital stay after surgery, pathological diagnosis, postoperative therapy, and survival data were reviewed from the electronic medical records library.

The study was approved by the Ethics Committee of the sixth medical center of the Chinese PLA General Hospital (No. 2019112507).

### Follow‐up

Follow‐up was performed every three months for each patient, and included physical status, and contrast computed tomography (CT) of the chest and upper abdomen. Contrast magnetic resonance imaging (MRI) of the brain and nuclear bone scan were performed every six months. Positron emission tomography (PET) scan was utilized if required.

Event‐free survival (EFS) was calculated from surgery to recurrence or progression, postoperative survival (POS) was calculated from surgery to death for any reason, and overall survival (OS) was calculated from first diagnosis to death for any reason.

## Results

### Patient characteristics

Between January 2016 and June 2019, 962 patients had undergone surgery for lung cancer. Among these, 13 patients had received preoperative targeted therapy. Four patients were ruled out of the analysis: three patients who had lost the opportunity of surgical treatment due to multiple pulmonary metastasis underwent wedge resection to secure intact histological and genomic information to enable guidance on further treatment; one patient had stage II disease at the time of first diagnosis. A total of nine patients were eventually enrolled into our study.

The baseline demographic and clinical characteristics of eligible patients are shown in Table 1. There were five males and four females with a median age of 56 years (range, 40–65 years). All patients were diagnosed with lung adenocarcinoma, staged from IIIa–IVb at the time of first diagnosis. Metastasis was found in the mediastinal lymph nodes, pleural membrane, lung and brain. Epidermal growth factor receptor (*EGFR*) mutation was discovered in eight patients and anaplastic lymphoma kinase (ALK) translocation was discovered in one patient. Corresponding targeted therapy agents included EGFR‐targeted agents (Erlotinib, Icotinib or Gefitinib) and ALK‐targeted agent (Crizotinib). The duration of targeted therapy before surgery ranged from two to 46 months.

**Table 1:** Characteristics of patients undergoing salvage surgery after targeted therapy

Case	Gender	Age	Histology	Clinical stage	Metastasis	Driver gene	Targeted therapy agent	Duration of targeted therapy	Other treatment	Therapeutic effect
1	Female	63	Ad	cT2N0M1a, IVa	Pleura effusion	*EGFR*, exon 19 deletion	Icotinib	46 months	None	PR
2	Male	51	Ad	cT2N3M0, IIIb	LNs	*EGFR*, exon 21 L858R	Gefitinib	12 months	six cycles of chemotherapy	PR
3	Male	45	Ad	cT2N0M1a, IVa	Pleura effusion	*EGFR*, exon 19 deletion	Gefitinib	six months	None	PR
4	Female	65	Ad	cT2N0M1b, IVb	lung	*EGFR*, exon 21 L858R mutation	Icotinib	six months	None	PR
5	Male	40	Ad	cT2bN3M1, IVb	LNs, lung	*EGFR*, exon 19 deletion	Icotinib	14 months	None	PR
6	Male	42	Ad	cT2N2M0, IIIa	LNs	*EGFR*, exon 21 L858R mutation	Tarceva	two months	None	SD
7	Male	57	Ad	cT2N2M0, IIIa	LNs	*EGFR*, exon 21 L861 mutation	Icotinib	three months	None	SD
8	Female	56	Ad	cT2N2M1a, IVa	LNs, pleura effusion	*ALK* translocation	Crizotinib	eight months	None	PR
9	Female	59	Ad	cT2N2M1b, IVb	LNs, brain	*EGFR*, exon 19 deletion	Icotinib	two months	Gamma knife for brain metastasis	SD

Ad, adenocarcinoma; ALK, anaplastic lymphoma kinase; EGFR, epidermal growth factor receptor; LNs, lymph nodes; PR, partial response; SD, stable disease.

No other anticancer therapy was administered to these patients, with the exception of case 2, who had received six cycles of chemotherapy, and case 9 who had received gamma knife therapy for brain metastasis. After targeted therapy, partial response (PR) was reached in six patients and stable disease (SD) was achieved in three patients, according to the preoperative assessment.

### Perioperative factors

As shown in Table 2, seven patients underwent salvage surgery because of residual tumor and two patients because of slight disease relapse.

**Table 2:** Perioperative factors of patients undergoing salvage surgery after targeted therapy

Case	Reason for salvage surgery	y‐stage	Interval (days)	Surgical procedure	Surgical accident	Operation time (minutes)	Intraoperative Blood loss (mL)
1	Relapse	T2bN0M0, IIa	9	(open) RUL + LND	Bleeding	130	840
2	Residual tumor	T2aN0M0, Ib	7	(VATS) LUL + LND	None	110	100
3	Relapse	T2aN0M0, Ib	14	(open) LUL + LND	Extensive pleural adhesion	170	250
4	Residual tumor	T2aN0M0, Ib	5	(VATS) RLL + LND	None	90	120
5	Residual tumor	T1bN0M0, Ib	7	(VATS) LUL + LND	None	110	100
6	Residual tumor	T2aN2M0, IIIa	7	(open) RUL + LND	Extensive pleural adhesion	150	220
7	Residual tumor	T2aN2M0, IIIa	7	(VATS) LUL + LND	None	90	100
8	Residual tumor	T1aN0M0, Ia	6	(VATS) RLL + LND	None	80	140
9	Residual tumor	T2aN2M0, IIIa	5	(VATS) RLL + LND	None	90	100

LND, lymph node dissection; LUL, left upper lobectomy; open, open surgery; RLL, right lower lobectomy; RUL, right upper lobectomy; VATS, video‐assisted thoracoscopic surgery; y‐stage, the clinical stage after targeted therapy.

The median duration of targeted therapy before surgery was six months (range, 2–46 months). Of note, there were three cases who received targeted therapy before salvage surgery for a relatively short duration. Case 6, a 42‐year‐old male patient, was assessed with unresectable lung adenocarcinoma in a county‐level hospital and received Tarceva therapy there because of *EGFR* mutation. After two months of observation, there was no obvious tumor shrinkage and the patient was transferred to our center. Reassessment found that the tumor was actually resectable even at the time of first diagnosis, and therefore the patient received salvage surgery in our center. Cases 7 and 9 were two patients with resectable tumor at the time of first diagnosis who all refused surgical treatment, only receiving targeted therapy at the start of treatment. After three and two months, respectively, they changed their minds because there had been no obvious response to targeted therapy and they subsequently received salvage surgery in our center.

All patients were reassessed for resectability. The interval between targeted therapy and surgery ranged from 5–14 days. Lobectomy procedures plus systematic lymph node dissection were performed on all nine patients, including six who underwent video‐assisted thoracoscopic surgeries (VATS). In three cases, operations had to be transferred from VATS to open surgery due to accidental bleeding (case 1) or extensive pleural adhesion (cases 3 and 6). The median operation time and intraoperative blood loss were 110 minutes (range, 80–170 minutes) and 120 mL (range, 100–840 mL), respectively.

### Postoperative factors

As shown in Table 3, there was one patient with a minor postoperative complication of atrial fibrillation and the postoperative complication rate was 11.1% (1/9). There was no in‐hospital death. The median time of hospital stay after surgery was five days (range, 4–8 days). Pathological examination on surgical specimens identified three patients whose pathological results were different from their preoperative results: one patient with mixed histological types (case 2) and two patients with gene mutation of *T790M* (case 3 and 7). A negative surgical margin was achieved in all cases. The postoperative pathological stages of four patients (cases 2, 3, 6 and 8) were confirmed to be different from their preoperative y‐stages, due to unexpected lymph node metastasis.

**Table 3:** Postoperative factors of patients undergoing salvage surgery after targeted therapy

Case	Postoperative complication	Hospital stay (days)	Histology	Pathological stage	Driver gene	Postoperative treatment	POS (months)	OS (months)
1	None	7	AD	T2bN0M0, IIa	*EGFR*, exon 19 deletion	Icotinib	44	89
2	None	5	SCC + AD+SC	T2aN2M0, IIIa	*EGFR*, exon 21 L858R mutation	Gefitinib, radiotherapy	30	42
3	None	8	AD	T2aN2M0, IIIa	*EGFR* exon 20, T790M mutation	Osimertinib	27	33
4	None	4	AD	T2aN0M0, Ib	*EGFR*, exon 21 L858R mutation	Icotinib, chemotherapy	19	25
5	None	5	AD	T1cN0M0, Ia	*EGFR*, exon 19 deletion	Icotinib	17	31
6	Atrial fibrillation	8	AD	T1cN1M0, IIa	*EGFR*, exon 21 L858R mutation	Icotinib	14	16
7	None	4	AD	T2aN2M0, IIIa	*EGFR*, exon 20, T790M mutation	Osimertinib	7	10
8	None	5	AD	T1aN2M0, IIIa	*ALK* translocation	Crizotinib	6	14
9	None	5	AD	T1aN2M0, IIIa	*EGFR*, exon 19 deletion	Icotinib	5	7

AD, adenocarcinoma; ALK, anaplastic lymphoma kinase; EGFR, epidermal growth factor receptor; OS, overall survival calculated from the first diagnosis; POS, post‐operative survival; p‐stage, pathological stage; SC, sarcomatoid carcinoma; SCC, squamous cell carcinoma.

### Survival

**Figure 1:**
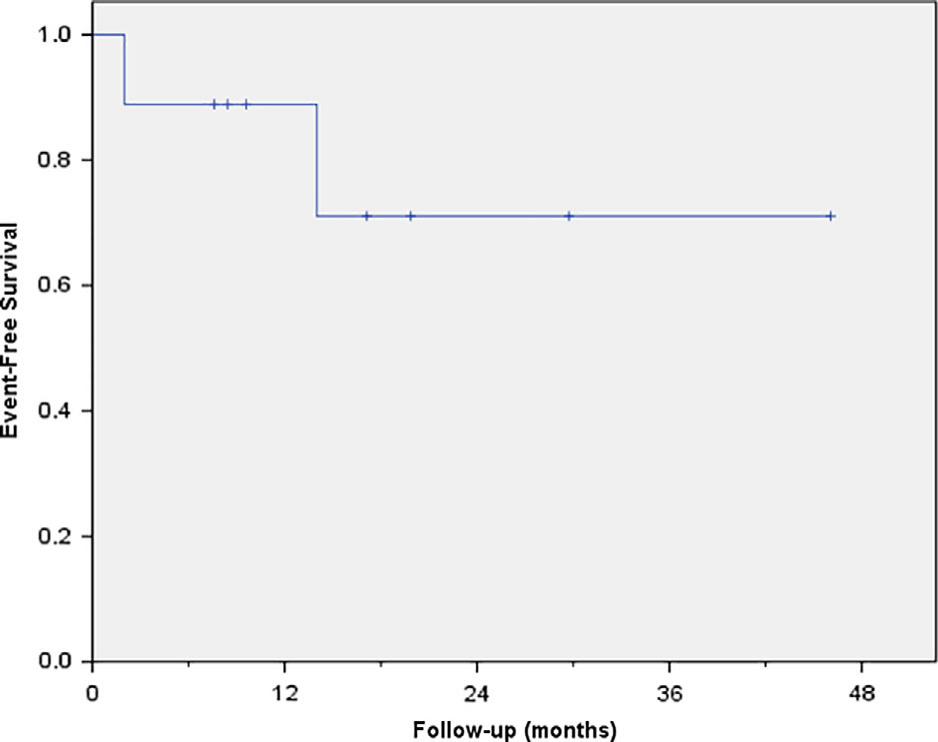
The event‐free survival curve.

All patients were alive at the time of the analysis. The median follow‐up time of patients in this study was 17 months (range, 5–44 months). There were two patients with disease progression and the median EFS was 14 months (range, 2–44 months) as shown in Figure 1. The median postoperative survival (POS) was 17 months (range, 5–44 months) and median overall survival (OS) was 25 months (range, 7–89 months), as shown in Table 3.

## Discussion

In this case series, we retrospectively analyzed the feasibility and efficiency of salvage surgery in patients with advanced lung cancer after targeted therapy. To the best of our knowledge, this is the first case series report on this subject.

In our analysis, we focused on patients with advanced (stage III or IV) NSCLC. It is our understanding that the treatment strategy for patients with early‐stage (stage I or II) NSCLC is relatively consistent (surgery is generally considered as the first choice) and the prognosis of patients is relatively satisfactory. By contrast, the prognosis of patients with relatively advanced NSCLC is far from satisfactory due to the complicated nature of the disease at this stage and unclarified treatment strategy. Therefore, we only included those patients with stage III or IV NSCLC and excluded those with early‐stage NSCLC.

Salvage surgery after effective targeted therapy gives full access to the advantages of surgery and targeted therapies. This is an idea of multidisciplinary therapy and individualized therapy.^26^ Similar to the role of surgery in neoadjuvant targeted therapy for lung cancer, the purpose of salvage surgery is to remove the residual or recurrent tumors when effective targeted therapy has transferred unresectable disease to resectable disease in some patients.^27^, ^28^ Moreover, an intact tumor specimen after salvage surgery can provide accurate and individual molecular pathological information in every patient. In our case 2, a mixed histological type of lung cancer was diagnosed, while in cases 3 and 7, *T790M* mutation was identified which is related to drug‐resistance of first‐generation agents targeting *EGFR* mutation.^29^ In concordance with the findings of Yoshida *et al*. these postoperative findings were key information which led to consequential treatment.^13^

Given the fact that the effect of targeted therapy is not static in one patient but will enter the plateau stage sooner or later,^4^, ^6^ we tended to carry out salvage surgery earlier (eg cases 2, 4, 5 and 8 in our series), in case the opportunity of surgical intervention was lost due to possible disease progression when drug resistance occurred over time. For patients who started with no apparent response to targeted therapy for more than two months, we held a similar view and suggested that they received salvage surgery if fit (eg cases 6, 7 and 9 in our series). There were also some patients receiving salvage surgery only when drug resistance of targeted therapy was proven by disease recurrence because they were afraid of surgical trauma and the inherent risks (eg cases 1 and 3 in our series). Since the number of cases reports in the literature is so small, whether salvage surgery should be carried out and how long is sufficient to judge the effect of targeted therapy is still inconclusive.

In terms of survival, the effect of salvage surgery is promising. All nine patients in our group were alive at the time of this analysis. After a mean follow‐up duration of 18.9 months, their median EFS reached 14 months, median POS 17 months, and OS reached 25 months. By contrast in the literature, the median progression‐free survival (PFS) of patients treated by targeted therapy with first generation drugs was only about 10.0 months and the PFS of patients who failed primary chemotherapy was only 3–6 months.^30^, ^31^, ^32^

Surgical trauma and risks are usually the major concerns of salvage surgery. The possible existence of lymph node metastasis, tumor invasion, pleural adhesion or destruction of hilar structures may make surgery more difficult. The physical status of patients may also deteriorate due to the side effects of preoperative treatment. However, according to our results, the process of salvage surgery is not so difficult or risky as imagined. Although pleural adhesion and fibrosis had developed in some patients in our group, radical surgery was achieved in all of them. There were six patients out of nine who received salvage surgery via minimally invasive thoracoscopic approaches. The intraoperative blood loss, operation time, postoperative hospital stay and postoperative complication rate in our group were similar to those for regular surgery. This was consistent with other reports on salvage surgery for lung cancer.^28^ Therefore, in our experience, it is feasible to perform surgery after targeted therapy. Undoubtedly, serious preoperative assessment on tumor resectability and patient fitness is essential to ensure feasibility and safety.^21^

Notably in our series there was a deviation between the preoperative clinical stage (y‐stage) and the postoperative pathological stage (p‐stage) in some patients, mainly due to the difference in lymph node status. The reason for this might be that preoperative targeted therapy had decreased the size and proportion of positive lymph nodes and thus the preoperative findings of PET‐CT or CT scan were not always consistent with the real status of the lymph nodes.^33^, ^34^ However, preoperative CT or PET‐CT is useful to assess the tumor resectability after targeted therapy.

The limitations of this study included the small sample size, its retrospective nature, the heterogeneity of baseline characteristics of enrolled patients, and the relatively short median follow‐up time. Given this, the results of our study should be interpreted with caution and it is hard to draw any definitive conclusion from our findings. However, this study highlighted salvage surgery as a feasible and promising therapeutic option for advanced NSCLC after targeted therapy in some selective fit patients. We look forward to future prospective studies to provide high‐level clinical evidence on this subject.

## Disclosure

All authors declare no conflict of interest.
